# High-Fat Diet and Age-Dependent Effects of IgA-Bearing Cell Populations in the Small Intestinal Lamina Propria in Mice

**DOI:** 10.3390/ijms22031165

**Published:** 2021-01-25

**Authors:** Yuta Sakamoto, Masatoshi Niwa, Ken Muramatsu, Satoshi Shimo

**Affiliations:** 1 Department of Physical Therapy, Faculty of Health Sciences, Health Science University, 7187 Kodachi, Fujikawaguchiko-machi, Minamitsuru-gun, Yamanashi 401-0380, Japan; y.sakamoto@kenkoudai.ac.jp; 2 Graduate School of Health Sciences, Kyorin University, 5-4-1 Shimorenjaku, Mitaka-shi, Tokyo 181-8612, Japan; 3 Department of Occupational Therapy, Faculty of Health Sciences, Kyorin University, 5-4-1 Shimorenjaku, Mitaka-shi, Tokyo 181-8612, Japan; mt-niwa@ks.kyorin-u.ac.jp; 4 Department of Physical Therapy, Faculty of Health Sciences, Kyorin University, 5-4-1 Shimorenjaku, Mitaka-shi, Tokyo 181-8612, Japan; k-muramatsu@ks.kyorin-u.ac.jp; 5 Department of Occupational Therapy, Faculty of Health Sciences, Health Science University, 7187 Kodachi, Fujikawaguchiko-machi, Minamitsuru-gun, Yamanashi 401-0380, Japan

**Keywords:** high-fat diet, mice, small intestine, lamina propria, Immunoglobulin A

## Abstract

Several studies highlighted that obesity and diabetes reduce immune function. However, changes in the distribution of immunoglobins (Igs), including immunoglobulin-A (IgA), that have an important function in mucosal immunity in the intestinal tract, are unclear. This study aimed to investigate the impaired immune functions in the context of a diet-induced obese murine model via the assessment of the Igs in the intestinal villi. We used mice fed a high-fat diet (HFD) from four to 12 or 20 weeks of age. The distributions of IgA, IgM, and IgG1 were observed by immunohistochemistry. Interestingly, we observed that IgA was immunolocalized in many cells of the lamina propria and that immunopositive cells increased in mice aged 12 to 20 weeks. Notably, mice fed HFD showed a reduced number of IgA-immunopositive cells in the intestinal villi compared to those fed standard chow. Of note, the levels of IgM and IgG1 were also reduced in HFD fed mice. These results provide insights into the impaired mucosal immune function arising from diet-induced obesity and type 2 diabetes.

## 1. Introduction

Obesity and Type 2 diabetes mellitus (T2DM) have become quite prevalent worldwide; according to the World Health Organization (WHO), the global prevalence of obesity has nearly tripled since 1975 and is a major risk factor for diabetes and other non-communicable diseases [1]. Globally, an estimated 463 million people had diabetes mellitus (DM) in 2019, and T2DM accounted for approximately 90% of all cases [2]. Thus, obesity and diabetes are among the most important medical challenges in the present. Moreover, obesity was defined as a risk factor for various (infectious) diseases, including COVID-19 [3,4], chronic inflammatory diseases such as cardiovascular disorders [5,6], asthma [7], and liver disease [8], and associated with worse outcomes [9,10]. However, the detailed mechanism of increased susceptibility to infection in the context of obesity or DM remains unclear [4,10].

The intestinal tract has a simple columnar epithelium essential for nutrient uptake and is constantly exposed to commensals and exogenous bacteria, thus serving as an organ of the immune system with a well-developed immune function. Of note, the gut microbiome present in the gastrointestinal tract maintains a symbiotic relationship with the host, supporting metabolism and promoting the maturation of gut immunity [11]. In addition, Immunoglobulin (Ig)-A, secreted into the lumen of the intestinal tract by plasma cells within the lamina propria (LP), plays a major role in mucosal immunity as the first line of defense protecting against intestinal toxins and pathogenic microorganisms [12]. Furthermore, certain members of the gut microbiome are involved in the differentiation of lymphocytes (e.g., Regulatory T-cell and Th 17 cell) and IgA production in the gut [11]. Additionally, the differentiation of IgA-secreting plasma cells is strictly regulated by T cells, which inhibit excessive inflammation due to gut bacteria and maintain gut homeostasis, balancing the complex interaction between gut immunity and the gut microbiome [11,13]. Therefore, to understand intestinal mucosal immunity, it is necessary to elucidate the function of Immunoglobulins (Igs), particularly of IgA.

The gut microbiota is altered by the lifestyle and dietary choices of the host and is associated with obesity and T2DM, in addition to impaired immune function [14,15,16,17]. In fact, one hypothesis to explain immune suppression caused by obesity and T2DM is that intestinal mucosal immunity dysfunction is associated with alterations in the gut microbiota. Because the biological structure reflects the function, the elucidation of changes in the intestinal pattern of IgA distribution has the potential to contribute to the understanding of the effects of obesity and diabetes on mucosal immunity. However, conventional preparation methods with respect to morphological specimens make it difficult to examine the distribution of soluble serum proteins due to artificial changes in histology, such as the diffusion of soluble proteins and antigen-masking [18,19]. Remarkably, the in vivo cryo-technique (IVCT), developed by Ohno et al. in 1996 [20], enables us to observe the distribution of in vivo soluble serum proteins in the intestinal tract with no artifacts using an isopentane propane liquid cryogen [21]. Of note, the results of a previous study comparing IgA immunoreactivity of intestinal villi with multiple fixation methods showed that the conventional fixation method did not detect immunoreactivity in the mucosal epithelium with diffuse staining around the intestinal crypts, whereas IVCT allowed the detection of strong plasmacytoid positive staining, including in the extracellular space [21]. Thus, IVCT may be superior to conventional fixation methods for the assessment of IgA-producing cells and their localization and distribution in the intestinal villi to evaluate intestinal immunity.

It has been reported that promoting factors of IgA secretion are decreased in high-fat-diet (HFD)-fed mice [22]. Another paper suggested that IgA secretion may be inhibited in the context of low-grade inflammation and metabolic syndrome in obese humans and mice [23]. However, most reviews on diabetes and immunity do not discuss humoral immunity, including IgA responses [24]. Only one review on T2DM and infectious diseases in humans reported a lack of humoral immunity [25]. Thus, it is possible that the intestinal immune function is impaired in the context of obesity and T2DM. In this study, we investigated the hypothesis that obesity and T2DM alter IgA distribution and decrease the population of IgA-bearing cells in the intestinal villi. In brief, because the symptoms of obesity and diabetes depend on disease duration, we examined the distribution of IgA in jejunal villi as well as the changes in IgA-bearing cells within the LP using IVCT in the context of optical microscopy, in mice fed with a high-fat diet for different periods.

## 2. Results

### 2.1. Validation of the T2DM Model via the Evaluation of the Body Weight and Blood Glucose Levels of Mice

**Figure 1:**
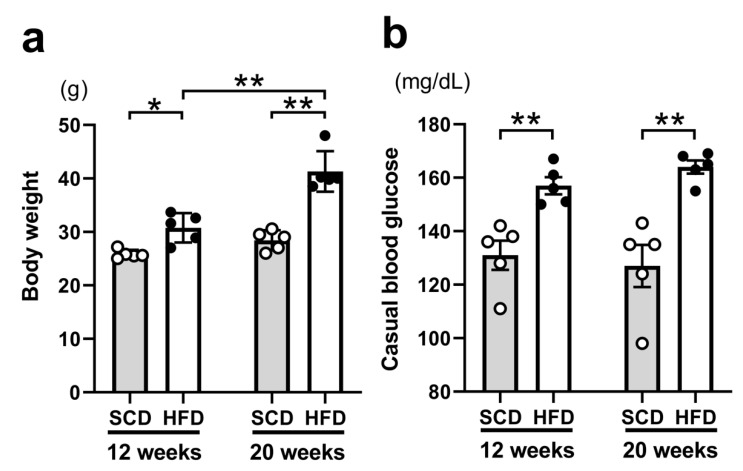
Comparison of the indicators of obesity and diabetes. Body weight (**a**). High-fat-diet (HFD)-fed mice showed a significantly higher body weight at 12 weeks compared to those fed a standard chow diet (SCD); the same was true at 20 weeks. Casual blood glucose levels (**b**). There were only significant differences in the diet models, with HFD resulting in significantly higher blood glucose levels at 12 and 20 weeks. The same number of animals was used in all groups (n = 5 per group). Results are presented as the mean  ± SEM. * *p*  <  0.05, ** *p*  <  0.01 (Two-way ANOVA with Holm-Šídák’s post hoc analysis).

To assess the appropriateness of our T2DM model, the body weight and casual blood glucose levels of mice were measured immediately before the experiments; the results are shown in Figure 1.

The body weight differed significantly depending on the diet (*p* < 0.001), age (*p* < 0.001), and interaction (*p* = 0.003). Furthermore, the post hoc analysis indicated significant differences in the context of the diet in both 12-week-old mice [standard chow diet (SCD) (25.8 ± 0.4 g) vs. HFD (30.8 ± 1.2 g), *p* = 0.023] and 20-week-old mice [SCD (28.4 ± 0.8 g) vs. HFD (41.3 ± 1.7 g), *p* < 0.001], and in the context of age between 12- and 20-week-old mice fed with HFD (*p* < 0.001; Figure 1a). Thus, mice fed HFD were overweight by the age of 12 weeks, and the degree of overweight increased in 20-week-old HFD-fed mice. In addition, the casual blood glucose levels were only significantly different between mice fed with different diets (*p* < 0.001); post hoc analysis revealed SCD (131.0 ± 5.5 mg/dL) vs. HFD (157.0 ± 3.2 mg/dL) at 12 weeks (*p* = 0.008) and SCD (127.0 ± 7.9 mg/dL) vs. HFD (164.0 ± 2.5 mg/dL) at 20 weeks (*p* < 0.001) as significantly different groups (Figure 1b). Thus, we confirmed that 12-week-old mice already showed HFD-induced hyperglycemia. Therefore, altogether, these results suggest that HFD contributes to obesity and hyper glycemia.

### 2.2. Comparison of Light Microscopy Images of HFD and SCD Fed Mice at Different Ages

Serial paraffin sections were prepared for both SCD and HFD-fed mice aged 12 and 20 weeks, stained with HE, as well as with antibodies anti-IgA, -IgM, and -IgG1, and imaged using a light microscope.

**Figure 2:**
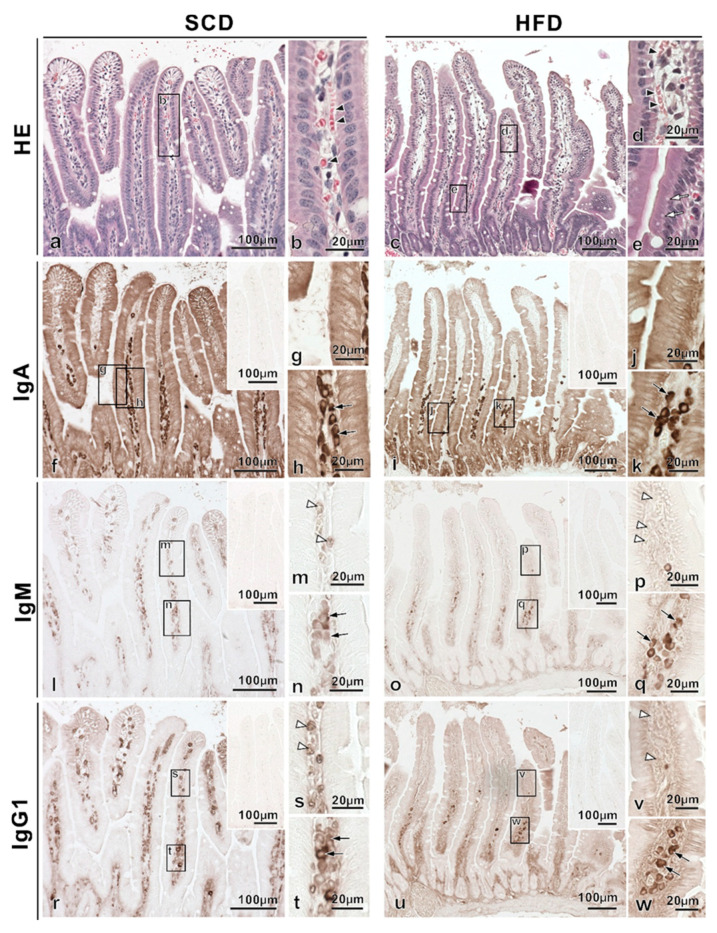
Comparison of the intestinal villi of standard chow diet (SCD) and high-fat-diet (HFD) fed 12-week-old mice. Paraffin-embedded tissue serial sections were obtained from each diet model. HE-staining of villi in the small intestines from SCD-fed mice showed regularly aligned simple columnar epithelium (**a**,**b**). HE-staining of tissues from SCD and HFD-fed mice showed red blood cells (arrowheads in **b**,**d**). HE-staining of tissue sections from HFD-fed mice revealed vacuoles in simple columnar epithelial cells (**c**, white arrows in **e**). IgA-immunoreactivity was detected in the cytoplasm of epithelial cells in SCD (**f**–**h**) and HFD-fed mice (**i**–**k**), and many IgA-immunopositive cells were also observed in the lamina propria of intestinal villi (black arrows in **h**,**k**). However, IgA-immunopositive cells in the lamina propria of intestinal villi in HFD-fed mice showed localization toward the bottom, with decreased immunopositivity compared to that in SCD-fed mice. IgM and IgG1 stainings showed common findings: Ig-immunopositive vessels (white arrowheads in **m**,**p,s,v**) and Ig-immunopositive cells (arrows in **n**,**q**,**t**,**w**) were observed. However, Ig-immunopositive vessels in HFD-fed mice were not immunopositive for both IgM and IgG1 (white arrowheads in **p**,**v**). The inset also indicates immuno-controls (**f**,**i**,**l**,**o**,**r**,**u**).

First, histological findings of villi in the small intestines (HE-staining) were compared among 12-week-old mice fed different diets (Figure 2a–e). Of note, via the observation of the regularly aligned simple columnar epithelium, we confirmed that there were no ice crystals. Moreover, red blood cells along the blood vessels, a characteristic of IVCT, were observed in HE-stained images (Figure 2b,d). Furthermore, the presence of vacuoles in simple columnar epithelial cells clearly distinguished HFD-fed mice from SCD-fed mice (Figure 2e). Then, the immunolocalization of IgA in the villi of small intestinal tissues of 12-week-old mice was examined in different specimens prepared with IVCT (Figure 2f–k); in both diets, IgA immunoreactivity was detected in the cytoplasm of epithelial cells (Figure 2g,j), and IgA-immunopositive cells were also observed in the LP of intestinal villi (Figure 2h,k). However, the IgA-immunopositive cells in the LP of intestinal villi were localized in the bottom of the LP in HFD-fed mice, with decreased immunopositivity, compared to that in SCD-fed mice. On the other hand, both IgM and IgG1 were present in Ig-immunopositive vessels (Figure 2m,s) in SCD-fed mice and in Ig-immunopositive cells (Figure 2n,q,t,w) in the two diet models. However, blood vessels in HFD-fed mice were not immunopositive for both IgM and IgG1 (Figure 2p,v), even at the same sites where blood vessels were confirmed by HE staining of serial paraffin sections.

**Figure 3:**
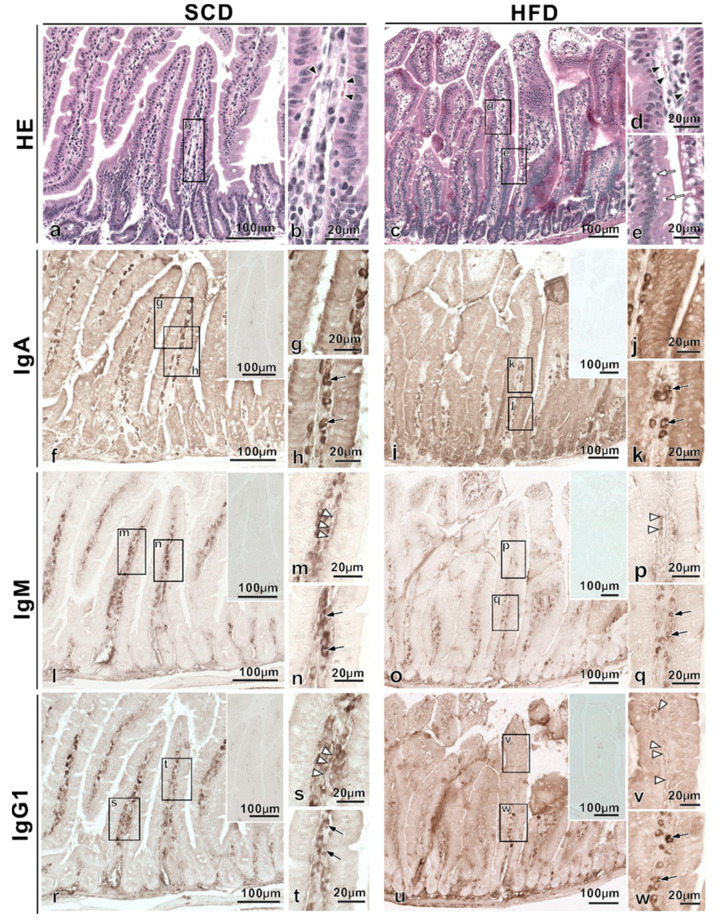
Comparison of the intestinal villi of standard chow diet (SCD) and high-fat-diet (HFD) fed 20-week-old mice. Paraffin-embedded tissue serial sections were obtained from each diet model. HE-staining of villi in the small intestines from standard chow diet (SCD)-fed mice showed regularly aligned simple columnar epithelium (**a**,**b**). HE-staining of tissue sections from high-fat-diet (HFD)-fed mice revealed vacuoles in monolayer columnar epithelial cells (**c**, white arrows in **e**). HE-staining of tissues from SCD and HFD-fed mice showed red blood cells (arrowheads in **b**,**d**). IgA immunoreactivity in SCD-fed mice was detected in the cytoplasm of epithelial cells (**g**), and many IgA-immunopositive cells were also observed in the whole lamina propria of intestinal villi (**f**, black arrows in **h**). Meanwhile, 20-week-old HFD-fed mice exhibited IgA immunoreactivity localized on the bottom of the lamina propria (**i**,**j**), and IgA-immunopositive cells (black arrows in **k**) were further reduced compared to those in SCD-fed mice. In both diet models, IgM and IgG1 immunoreactivity were observed in blood vessels (white arrowheads in **m**,**s**,**p**,**v**) and Ig-immunopositive cells (arrows in **n**,**q**,**t**,**w**). The inset also indicates immuno-controls (**f**,**i**,**l**,**o**,**r**,**u**).

Additionally, histological findings were compared among 20-week-old mice fed different diets as above (Figure 3). HE of 20-week-old mice revealed red blood cells, characteristic of IVCT, similar to 12-week-old mice (Figure 3b,d). Of note, the vacuoles observed in 12-week-old HFD-fed mice were even more pronounced in 20-week-old HFD-fed mice (Figure 3e). Additionally, IgA immunoreactivity in the cytoplasm of epithelial cells was widely detected in 20-week-old SCD-fed mice compared to 12-week-old SCD-fed mice (Figure 3g), and many IgA-immunopositive cells were also observed from the bottom to top of LP in intestinal villi (Figure 3f, arrows in 3h). Meanwhile, IgA immunoreactivity in 20-week-old HFD-fed mice was localized to the bottom of the LP (Figure 3j), with a reduced population of IgA-immunopositive cells (Figure 3i, arrows in 3k), compared to that in 12-week HFD-fed mice. Conversely, IgM and IgG1 immunoreactive blood vessels (Figure 3p,v) and Ig-immunopositive cells (Figure 3q,w) were similar between 20- and 12-week-old HFD-fed mice; however, the blood vessels were slightly more immunoreactive in 20-week-old HFD-fed mice than in 12-week-old HFD-fed mice (Figure 2p,v). The above findings were semi-quantified and are summarized in Table 1.

### 2.3. Comparison of the Number of Ig-Immunopositive Cells in the LP

**Figure 4:**
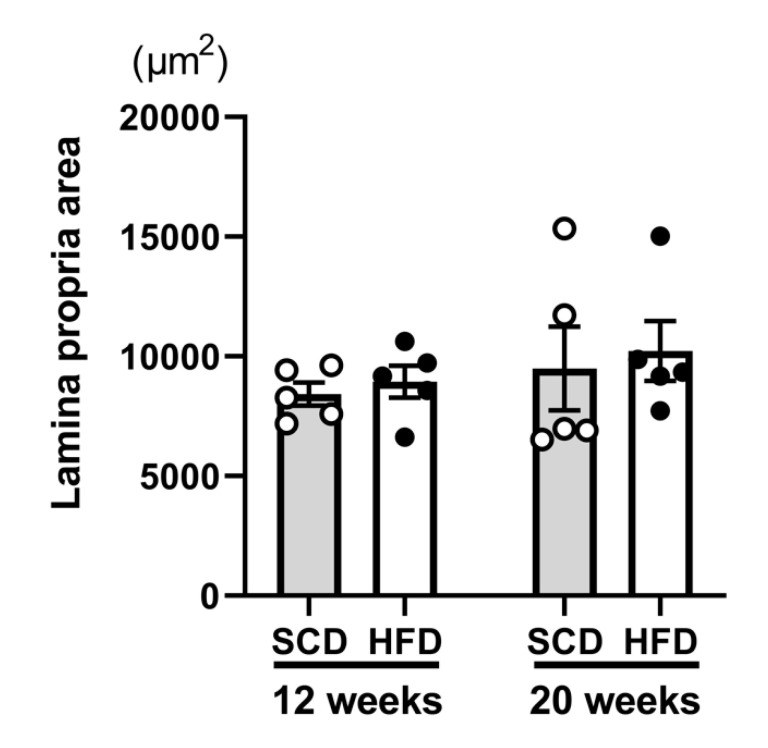
Comparison of the areas of the lamina propria used in quantitative analyses. There were no differences in the areas of the lamina propria analyzed either between the age groups (12-week and 20-week) or between the diet models (standard chow diet and high-fat diet) (Two-way ANOVA). The same number of animals was used in all groups (*n* = 5 per group).

**Figure 5:**
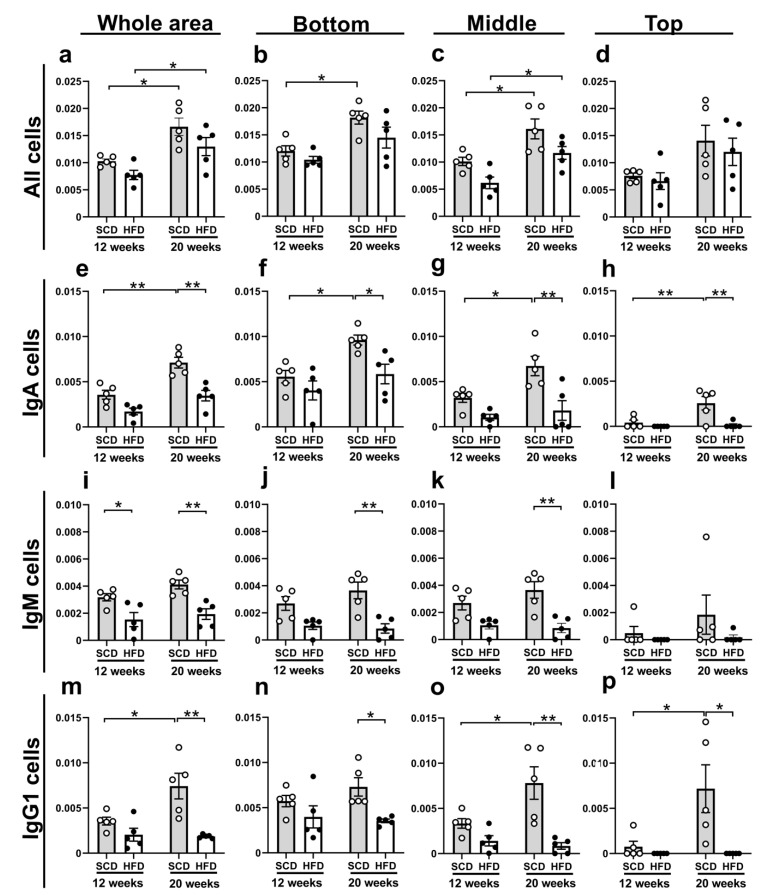
The number of Ig-immunopositive cells in the lamina propria of small intestinal villi. All cells in the whole lamina propria in the small intestinal villi were significantly different between 12-week and 20-week-old SCD-fed mice and between 12-week and 20-week-old HFD-fed mice (**a**). This trend was similar for all cells in the bottom (**b**) and middle (**c**) sections, but not for the top section (**d**). IgA-immunopositive cells in the whole area (**e**) were significantly different between 12- and 20-week-old SCD-fed mice and between SCD and HFD-fed 20-week-old mice. This trend was confirmed in all IgA-immunopositive cell comparisons, in the bottom (**f**), middle (**g**), and top (**h**) sections. IgM-immunopositive cells were significantly different between HFD and SCD-fed 12-week and 20-week-old mice in the whole area (**i**). This trend was similar for in 20-week-old mice on the bottom (**j**) and middle (**k**) sections, but the top (**l**) section did not differ significantly between the diet models. IgG1-immunopositive cells in the whole area (**m**) were significantly different between 12- and 20-week-old SCD-fed mice, and between SCD- and HFD-fed 20-week-old mice. Similarly, a significant difference in the distribution of IgG1-immunopositive cells was found in the middle (**o**) and top (**p**) sections. However, in the bottom section, a significant difference was found only between 20-week-old SCD-fed and HFD-fed mice (**n**). For the sake of comparison, we defined the central part of the villi as per a method of villi division detailed in the Quantitative analysis sub-section of the Materials and Methods. The same number of animals was used in all groups (*n* = 5 per group). Results are presented as the mean  ±  SEM of the number of cells per μm^2^. * *p*  <  0.05, ** *p* <  0.01 (Two-way ANOVA with Holm-Šídák’s post hoc analysis).

To disclose the differences in the distribution of Ig-immunopositive cells in the intestinal villi, we counted Ig-immunopositive cells in the entire or in specific areas of the LP. Overall, no significant differences were found between the different age groups (*p* = 0.32) or diet models (*p* = 0.59) with respect to the areas of the LP considered (Figure 4). All of the comparative results (number of cells per µm^2^) are shown in Figure 5.

HE-staining was used to assess all cells within the LP. The number of cells in the whole LP area was significantly different between the different age groups (*p* < 0.001) and diet models (*p* = 0.025). Furthermore, the post hoc analysis revealed significant differences between 12- and 20-week-old SCD-fed mice (*p* = 0.013) and between 12- and 20-week-old HFD-fed mice (*p* = 0.04), as shown in Figure 5a. This trend was similar for all cells in the bottom (12-week vs. 20-week in SCD; *p* = 0.017, Figure 5b) and middle (12-week vs. 20-week in SCD; *p* = 0.021, 12-week vs. 20-week in HFD; *p* = 0.030, Figure 5c) sections. However, the whole cells at the top were not significantly different (Figure 5d). Thus, these results suggest that the number of cells in the intestinal villi increased from 12 weeks of age to 20 weeks of age in SCD- and HFD-fed mice, but HFD-fed mice tended to have lower cell numbers than those in SCD-fed mice.

Additionally, at all ages and in the different diet models, the total numbers of Ig-immunopositive cells were higher with respect to IgA, followed by IgG1, and IgM. Of note, the number of Ig-immunopositive cells tended to be higher in SCD-fed mice than in HFD-fed mice. Importantly, the number of IgA-immunopositive cells in the whole LP area was significantly different between the age groups (*p* < 0.001) and diet models (*p* < 0.001); post hoc analysis demonstrated significant differences in the number of IgA-immunopositive cells between 12- and 20-week-old SCD-fed mice (*p* < 0.001) and between SCD-fed and HFD-fed 20-week-old mice (*p* < 0.001), as shown in Figure 5e. This trend was confirmed in all IgA-immunopositive cell comparisons: bottom (12-week vs. 20-week in SCD; *p* = 0.023; HFD vs. SCD in 20-week; *p* = 0.030, Figure 5f), middle (12-week vs. 20-week in SCD; *p* = 0.031, HFD vs. SCD in 20-week; *p* = 0.003, Figure 5g), and top (12-week vs. 20-week in SCD; *p* = 0.007, HFD vs. SCD in 20-week; *p* = 0.003, Figure 5h). Thus, these results suggest that the number of IgA-immunopositive cells in the LP increased between the ages of 12 and 20 weeks in the SCD-fed mice; however, there was no increase observed in HFD-fed mice.

The number of IgM-immunopositive cells in the whole LP area was also significantly different between the diet models (*p* < 0.001). Furthermore, the post hoc analysis demonstrated significant differences in the number of IgM-immunopositive cells between 12-week-old HFD and SCD fed mice (*p* = 0.033) and between 20-week-old HFD and SCD-fed mice (*p* = 0.005), as shown in Figure 5i. In addition, significant differences in the distribution of IgM-immunopositive cells between 20-week-old HFD and SCD-fed mice were also observed in the bottom (*p* = 0.003, Figure 5j) and middle (*p* = 0.003, Figure 5k) sections.

Last but not least, the number of IgG1-immunopositive cells in the whole LP area was significantly different between age groups (*p* = 0.040) and diet models (*p* < 0.001); the post-hoc analysis demonstrated significant differences between 12- and 20-week-old SCD-fed mice (*p* = 0.017) and between 20-week-old SCD-fed and HFD-fed mice (*p* = 0.001), as indicated in Figure 5m. Furthermore, significant differences in the distribution of IgG1-immunopositive cells were found between the middle (12-week vs. 20-week in SCD; *p* = 0.024, HFD vs. SCD in 20-week; *p* < 0.001, Figure 5o) and top (12-week vs. 20-week in SCD; *p* = 0.016, HFD vs. SCD in 20-week; *p* = 0. 011, Figure 5p) sections. However, in the bottom section, a significant difference was found only between 20-week-old SCD-fed and HFD-fed mice (*p* = 0.041, Figure 5n).

## 3. Discussion

In the present study, we examined changes in the distribution of Ig immunoreactive cells in the small intestinal villi using a mouse model of high-fat diet-induced T2DM. It is difficult to observe the distribution of soluble serum proteins, such as Igs, by conventional fixation methods used for morphological studies. In the present study, however, we were able to observe Igs in the small intestinal villi using immunohistochemistry after tissue fixation with IVCT.

In addition to the HFD-induced mice used in this study, various genetically deficient mice exist as animal models of T2DM. However, because many T2DM humans develop the disease due to diet options rather than due to genetic factors, diet-induced obesity-induced T2DM is the most appropriate disease model for our research context [26]. In this study, body weight and blood glucose levels at any time were used as indicators; importantly these indicators were significantly higher in HFD- vs. SCD-fed mice. However, glucose tolerance and insulin resistance tests were not performed to exclude the effect on fasting and glucose administration, because the fasting and refeeding affect mucosal immunity [27]. Of note, a previous study found abnormal glucose tolerance, insulin resistance, and increased lipid percentage after 12 weeks of high-fat diet administration, and reported prediabetic/early diabetes in combination with retinal findings [28]. Moreover, with respect to liver enzymes, alanine aminotransferase was significantly increased after about 10 weeks of HFD feeding [29]. Meanwhile, because diet-induced type 2 diabetic mice are pre-diabetic, with insulin resistance, a new model to investigate insulin deficiency in late type 2 diabetes has been proposed [30,31]. This said, in summary, our results strongly suggest the development of pre/early diabetes, symptoms that precede extreme hyperglycemia, and mimic the development of type 2 diabetes in humans. However, most of the dietary choices in the protocols used for diet-induced T2DM or obesity models are determined by the % kcal fat; the same was true for the diets used in this study [26]. Of note, HFD is also a high caloric diet, but there are few studies of HFD induced T2DM or obesity. Therefore, the debate over whether HFD induced T2DM or obesity phenotypes originate from a high-fat or high-calorie diet remains an issue.

An interesting finding of this study was that the number of whole cells and Ig-immunopositive cells in the LP of the small intestinal villi increased in SCD-fed mice from 12 to 20 weeks of age. In particular, the results of this study suggest that the number of IgA-immunopositive cells, the most important with respect to mucosal immunity in the intestinal mucosa, usually increase after 12 weeks of age. The C57BL/6J mice used in this study grew rapidly until 12 weeks of age and were considered adults from 12 to 24 weeks of age; aging began after 24 weeks of age [32]. Moreover, although there are previous studies describing changes in B-cell numbers in the jejunal mucosal endosymbiotic layer in infant [33] and aging mice [34,35], we did not find previous studies on the number of IgA-bearing cells in the jejunal villi of mice at the ages used in this study. Notably, the expression of the *RAG-1* gene in bone marrow B-cell precursors, such as the progenitor cells that give rise to IgA-secreting plasma cells, peaks at approximately 8–20 weeks in mice [36]. Therefore, as circumstantial evidence, the increased levels of normal IgA-bearing cells in mice between the ages of 12 and 20 weeks are consistent with the results of previous studies.

In general, B cells are classified into two sub-types, B1 and B2 cells. B2 cells constitute the majority of B cells in vivo, differentiate in a T cell-dependent manner in secondary lymphoid tissues, and produce specific antibodies [37]. On the contrary, B1 cells are known to make up a large percentage of B cells in the abdominal and thoracic cavities and are responsible for early immune responses, the suppression of commensal bacteria, and the production of innate IgM [34,37]. As mentioned above, IgA plays an important role in intestinal mucosal immunity, and it may either be produced by B2 cells via T cell-dependent pathways occurring in the Peyer’s patches, or by B1 cells via T cell-independent pathways in the intestinal LP [38]. Of note, previous studies have reported a T-cell-independent pathway predominantly involving B1-derived IgA [38]; however, B1-derived IgA alone cannot compensate for reduced IgA production [39]. Therefore, the current knowledge suggests that both pathways contribute to IgA secretion in intestinal mucosal immunity.

In the present study, a significant increase was observed in IgA-immunopositive cells between the ages of 12 and 20 weeks in SCD-fed mice; additionally, the population of IgA-immunopositive cells in 20-week-old HFD mice was significantly reduced compared to that in the SCD counterparts. These results suggest that HFD suppressed the response that normally increases the number of IgA-bearing cells in 12- to 20-week-old mice. Although not quantitatively analyzed, IgA-immunoreactivity in the cytoplasm of epithelial cells of intestinal villi was reduced by HFD, as revealed by light microscopy images. These findings suggest a decline in the immune function in the intestinal tract, making it more susceptible to infections. In contrast, a previous study examining the number of IgA-positive cells in the intestinal tract by flow cytometry using a mouse model of diabetes concluded that IgA-bearing B cells and plasma cells in the distal small and large intestines were not altered by HFD [22]. This report differs from the present study, probably because we only investigated the intestinal villi rather than the whole LP; of note, here, the distribution of IgA resembled that of the “living animal morphology” since we used IVCT. In line with the above, different studies examining IgA secretion in the intestinal tract showed different results [22,40]; therefore, it is difficult to make a general comparison.

Meanwhile, the number of IgM-immunopositive cells was significantly reduced in 20-week-old HFD-fed mice compared to 20-week-old SCD-fed mice. The present study did not identify any sub-types of B cells and, therefore, did not investigate whether the effect of HFD was T-cell dependent or independent. However, both IgA or IgG1 and IgM were reduced by HFD, indicating the possibility that both T-independent and T-cell-dependent pathways were involved in the process. Moreover, IgM has the same potential for complement activation and microbial clearance as those of IgG [41]. Thus, the decrease in immune function due to reduced IgM not only implies a reduction in IgA precursors, but also in immune function due to the increased intestinal permeability caused by HFD [41,42]. Furthermore, IgG1 is involved in the induction of complement activity and inflammation, and together with IgA and IgM, IgG modulates the symbiosis of the gut microbiome [41]. Therefore, the decrease in IgG1 populations in this study, suggests a decrease in the intestinal immune function as well as a decrease in IgA.

In this study, we examined the distribution of Ig-positive cells in the intestinal lamina propria induced by HFD-fed mice. Of note, there are several reports on dysfunctions related to the intestines caused by high-fat diets and hyperglycemia. For instance, regarding immunity, there are reports of the hyperglycemia-induced impairment of intestinal epithelial barrier function, innate immunity, and cellular immunity [24,42]. There are also reports of changes in the neurons of the myenteric plexus and in intestinal motility due to the effects of HFD-feeding [43,44]. Therefore, obesity and T2DM may have various effects on intestinal function, including the decrease of IgA responses here reported in the context of HFD-feeding.

There are several limitations to this study. First, our findings are limited to the small intestinal villi; this study did not examine changes in Igs in multiple organs of the intestinal immune system (e.g., Peyer’s patches, crypt patches, mesenteric lymph nodes, spleen). Second, the method used was limited to paraffin-embedded tissue sections, which does not fully explain the changes in the number of Ig-positive cells in all of the villi spaces. In addition, we used a single mouse model, the obesity-induced T2DM model; therefore, the effects of non-caloric factors cannot be ruled out. Further studies are needed to provide detailed insight into the mechanism behind the decline of immune function in the context of obesity and diabetes. Moreover, we were not able to disclose whether the observed changes in Ig-positive cells were due to the high-fat diet originated from hyperglycemia and insulin excess (T2DM) or from excessive lipid intake (obesity). Last but not least, we were also not able to verify whether the underlying gut microbiome was synchronized with the changes in Ig-positive cells. Further studies are needed to clarify the above questions in the future. However, since the distribution of Ig-positive cells within the intestinal villi can be effectively reproduced by IVCT, the results of this study may represent a distinctive finding with biological relevance.

## 4. Materials and Methods

### 4.1. Animals

Animal experiments were approved by the Health Science University Animal Care and Use Committee. C57Bl/6J mice (Charles River Laboratories, Wilmington, MA, USA, via Oriental Yeast Co., Ltd., Tokyo, Japan) were housed within single cages in a ventilated incubator at the Health Science University under 12-h light/dark cycles (light on from 07:00 a.m. to 07:00 p.m.), and a temperature of approximately 23 ± 1 °C. Food and water were provided ad libitum. Of note, four-week-old male mice were assigned to one of the following four groups (n = 5 per group): group 1—mice fed SCD (13% fat, 63% carbohydrates, 24% protein, 4.2 kcal/g; Lab Supply Inc., Fort Worth, TX, USA) from four to 12 weeks of age (12-week-old SCD); group 2—mice fed SCD from four to 20 weeks of age (20-week-old SCD); group 3—mice fed HFD (60% fat, 20% carbohydrates, 20% protein, 5.2 kcal/g; Research Diet, New Brunswick, NJ, USA) from four to 12 weeks of age (12-week-old HFD); and group 4—mice fed HFD from four to 20 weeks of age (20-week-old HFD). Immediately prior to the experiments, the body weight and casual blood glucose levels of the mice were measured to confirm obesity and diabetes. The casual blood glucose levels were measured with blood from the tail vein using a blood glucose meter (ACCU-CHEK Aviva, Roche DC Japan Co., Ltd., Minato, Tokyo, Japan).

### 4.2. “In Vivo Cryotechnique (IVCT)” for the Observation of the Small Intestine of Living Mice

Mice were anesthetized via injection of a mixture of medetomidine hydrochloride (0.75 mg/kg, Nippon Zenyaku Kogyo Co., Ltd., Koriyama, Fukushima, Japan), midazolam (4 mg/kg, Sandoz Co., Ltd., Minato, Tokyo, Japan), and butorphanol tartrate (5 mg/kg, Meiji Seika Pharma Co., Ltd., Chuo, Tokyo, Japan) at a dosage of 0.1 mL/10 g body weight. Of note, fasting was not performed to effect on immunoglobulin localization in the intestinal tract. Small intestine tissues, 2–3 cm from the end of the stomach, were exposed outside the body under normal blood circulation and carefully put on aluminum sheets in their abdominal cavities. Since artifacts usually appear farther away from the contact point of the cryogen in IVCT, the luminal side of the jejunum was exposed by an incision along the long axis of the intestine. IVCT was immediately performed via direct pouring of isopentane–propane cryogen (−193 °C), precooled in liquid nitrogen, over the small intestines from the luminal side. Liquid nitrogen was additionally poured over the frozen small intestines, which were then carefully taken out. Of note, IVCT in the context of the intestine was carried out as previously reported [21].

### 4.3. Freeze-Substitution and Paraffin-Embedding

The freeze-substitution solution consisted of absolute acetone containing 2% paraformaldehyde. The frozen small intestine tissues were freeze-substituted at approximately −80 °C in dry ice-acetone for 48 h and then put into a freezer at −30 °C for 2 h and at −10 °C for 2 h. Tissues were then refrigerated at 4 °C for 2 h and finally kept at room temperature for 1 h. Finally, tissues were washed with pure acetone, immersed in xylene, and routinely embedded in common paraffin wax.

### 4.4. Hematoxylin-Eosin (HE) Staining and Immunohistochemistry

All paraffin-embedded tissues were cut into 5-μm thick serial paraffin sections and mounted on Matsunami Adhesive Slide coated glass slides (Matsunami Glass, Kishiwada, Osaka, Japan). The thin sections were de-paraffinized with xylene and rehydrated in a graded series of ethanol and distilled water. Some sections were routinely stained with HE to observe the tissue morphology in the context of frozen conditions using a light microscope. After that, the sections were processed for immunohistochemistry analyses and incubated with 0.3% hydrogen peroxide in phosphate-buffered saline (PBS) and 2% gelatin in PBS for 1 h each. The sections were then immunostained via incubation overnight with different primary antibodies in 2% gelatin (from cold water fish skin, Sigma-Aldrich Co. LLC., St. Louis, MO, USA) at 4 °C. The primary antibodies used were goat or rabbit polyclonal antibodies against murine IgA (heavy chain of IgA, 1/9000 dilution, BETYL Inc., Montgomery, TX, USA, A90-103A), IgM (heavy chain of IgM, 1/2000 dilution, BETYL Inc., Montgomery, TX, USA, A90-102A), and IgG1 (heavy chain of IgG1, 1/2000 dilution, BETYL Inc., Montgomery, TX, USA, A90-105A). The immunocontrols were thin sections incubated in 2% gelatin without primary antibodies. Then, the immunostained sections were incubated with horseradish peroxidase-conjugated donkey anti-goat (Goat IgG whole molecule, 1/400 dilution, Abcam Inc., Cambridge, MA, USA, ab6667) for IgA and -IgG1 or goat anti-rabbit IgG (Rabbit IgG whole molecule, 1/400 dilution, Abcam Inc., Cambridge, MA, USA, ab7171) for IgM, at room temperature for 1 h and finally visualized with diaminobenzidine (DAB) in a buffer solution containing hydrogen peroxide for 3 or 5 min. The sections were observed as optical microscopic images under an all-in-one microscope (BZ-X800; KEYENCE, Osaka, Osaka, Japan, objective magnification 4× to 40×). Of note, the development of brownish cellular colors after reaction with DAB was defined as immunopositivity.

### 4.5. Quantitative Analysis

The distribution of Igs in the villi of the jejunum was assessed based on the quantification of cells in the optical microscopy images. The jejunal villi were determined to be centrally located when a single layer of columnar epithelium and central lacteal vessels were observed, and cells in the LP were counted. The images obtained were processed using ImageJ and Fiji plugins (https://fiji.sc/wiki/index.php/Fiji), and the LP were manually traced. In addition, to verify the differences in the distribution of cells within the villi, they were divided into three sections using the following procedure. First, two points where the simple column epithelium on both sides of the villi failed (ex; border of the crypt) were connected to obtain a baseline. Second, the point of intersection of the villi with the parallel line to the baseline, furthest from the baseline, was set as the tip. Third, the line connecting the tip and the midpoint of the baseline was set as the axis. Finally, the axis was divided into three parts, top, middle, and bottom from the tip. This method is based on that used in a previously published study in which the chorionic villi were divided into three sections [45]. Furthermore, for the sake of comparative quantification, the number of cells counted was normalized as the number of cells per µm^2^, either considering the whole LP, or divided sections.

### 4.6. Statistical Analysis

Statistical analysis was performed using the two-way ANOVA followed by Holm-Šídák’s post hoc analysis, because there were four groups with different ages (12 and 20 weeks) and diet (SCD and HFD). The GraphPad Prism (version 9; GraphPad Software., San Diego, CA, USA) was used to conduct statistical analyses. A *p*-value < 0.05 was considered statistically significant. Unless otherwise specified, variables were expressed as the mean ± standard error of the mean (SEM).

## 5. Conclusions

In the present study, tissue fixation with IVCT confirmed the distribution and structure of Ig-positive cells in the context of the “living animal morphology of small intestinal villi” in SCD- and HFD-fed mice. The results suggest that IgA normally increases in the LP (in intestinal villi) of SCD-fed mice between the ages of 12 and 20 weeks. However, HFD feeding suppresses the increase in IgA-immunopositive cells in the intestinal villi. Altogether, our results provide insights into the impaired mucosal immune function in diet-induced obesity and T2DM from the biological perspective.

## Figures and Tables

**Table 1:** Semi-quantification of Immunoglobulins in small intestinal villi.

			12-Weeks			20-Weeks	
		Ep	Lp	Bv	Ep	Lp	Bv
IgA	SCD	++	++	-	+++	+++	-
	HFD	+	+	-	+	+	-
IgM	SCD	-	++	++	-	++	++
	HFD	-	+	-	-	+	±
IgG1	SCD	-	++	++	-	++	++
	HFD	-	+	-	-	+	±

(+++) strong, (++) medium, (+) weak, (±) slightly, (-) negative. Ep: Epithelium, Lp: Lamina propria, Bv: Blood vessels. Strong: IgA-positive cells of SCD-fed 20-week-old mice showed in Figure 3f,h (black arrows). Medium: IgA-positive cells of SCD-fed 12-week-old mice showed in Figure 2f,h (black arrows). Weak: IgA-positive cells of HFD-fed 12- and 20-week-old mice showed in Figure 2i,k and Figure 3i,k (black arrows). Slightly, IgM and IgG1 immunoreactivity in BV in HFD-fed 20-week-old mice showed in Figure 3p,v (white arrowheads). Negative: non-immunoreactivity.

## Abbreviations

Ig	Immunoglobins
IgA	Immunoglobulin A
HFD	High fat diet
IgM	Immunoglobulin M
IgG1	Immunoglobulin G1
T2DM	Type 2 diabetes mellitus
WHO	World Health Organization
DM	Diabetes mellitus
COVID-19	Coronavirus disease 2019
LP	Lamina propria
IVCT	In vivo cryotechnique
SCD	Standard chow diet
HE	Hematoxylin-eosin
PBS	Phosphate-buffered saline
